# A Rare Case of Vascular Malformation in India: Cutis Marmorata Telangiectatica Congenita

**DOI:** 10.7759/cureus.64408

**Published:** 2024-07-12

**Authors:** Souvik Manna

**Affiliations:** 1 Community Medicine, Employees' State Insurance Corporation (ESIC) Medical College & Hospital, Alwar, IND

**Keywords:** cutis marmorata telangiectatica congenita, genetic disorders, congenital abnormality, venous malformations, vascular malformations, cmtc

## Abstract

The current case report presents a male baby, second born to nonconsanguineous parents at 38 weeks of gestation by lower segment cesarean section, with engorged blood vessels and distinctive patterns of discoloration and dilation of blood vessels on the left leg. A Doppler of the femoral artery and vein showed normal triphasic flow and waveforms without any evidence of significant luminal stenosis. There was also a lower limb length discrepancy of 1.5 cm. Genetic testing using fluorometric enzyme immunoassay screening revealed a negative screening report. Otologic screening using distortion product otoacoustic emissions revealed normal functioning of outer hair cells in both ears. The case was diagnosed as cutis marmorata telangiectatica congenita (CMTC), after ruling out genetic diseases. It was not associated with any other significant health problems. The diagnosis of CMTC was based on the appearance of the skin at birth, which became more noticeable shortly after two days. In this case, no specific treatment was warranted and the condition improved with time.

## Introduction

Cutis marmorata telangiectatica congenita (CMTC) is a rare vascular disorder that is present at birth (congenital). It primarily affects the skin and blood vessels, leading to a distinctive appearance of the skin. The condition is characterized by a marbled or mottled pattern on the skin, resembling a net or lace-like appearance. This is due to dilated blood vessels (telangiectasias) under the skin. The pattern may become more prominent when the skin is exposed to cold conditions, which differentiates it from physiological cutis marmorata, in which skin lesions typically resolve with warming.

CMTC commonly affects the limbs, particularly the legs, but it can occur in other parts of the body as well. It is usually unilateral but can be bilateral also. While the skin is primarily affected, CMTC can sometimes involve deeper blood vessels, leading to potential complications such as limb length discrepancy, muscle atrophy, or other developmental issues. Usually, the limb with lesions is longer than the normal limb because of increased blood flow. The severity of CMTC can vary widely. In some cases, it may be a minor cosmetic concern, while in others, it might be associated with more significant medical problems, like liver and kidney abnormalities. Most cases of CMTC occur sporadically, and two-thirds of the approximately 300 cases have been reported in women [1]. The current case is unique as it affected a male child, with no other malformations.

## Case presentation

**Figure 1:**
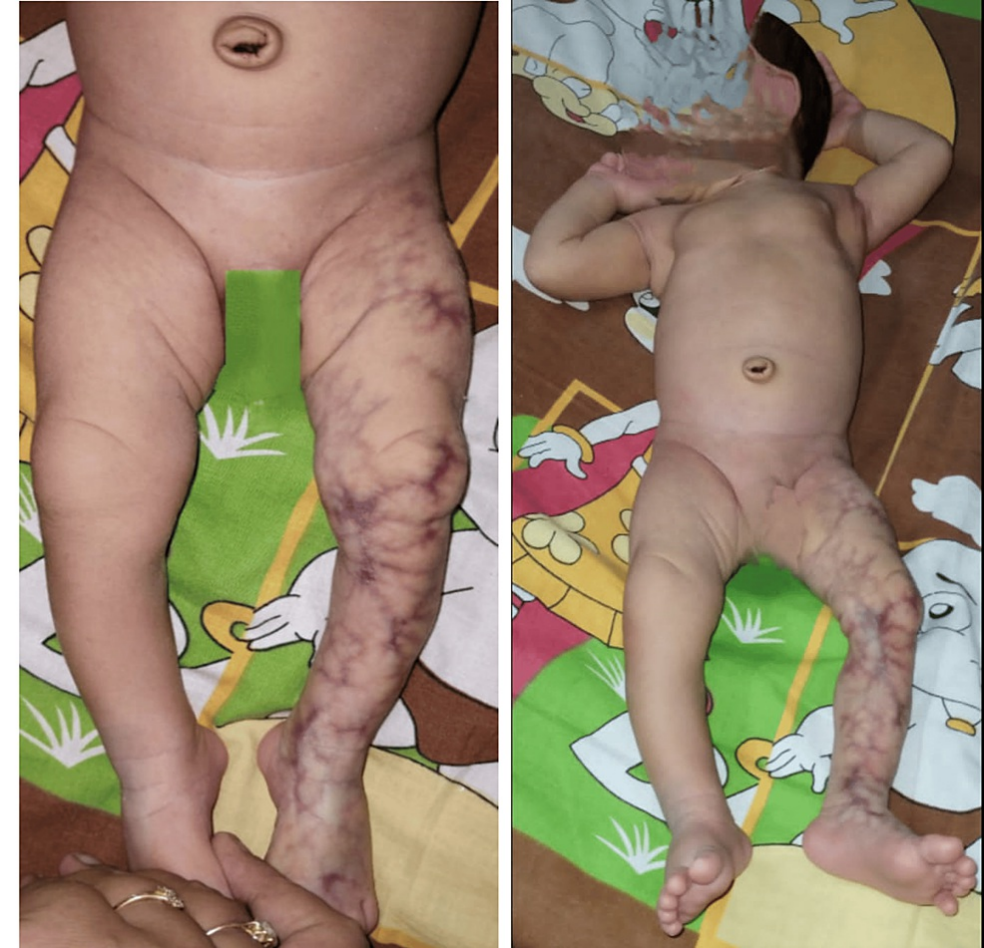
Clinical photographs showing engorged blood vessels on the left lower limb of the baby.

Written informed consent for the publication of clinical details and clinical images was obtained from the parents of the patient, and ethical approval was obtained as per the policies of the institute. A male baby, second born to nonconsanguineous parents (i.e., parents not related by blood) at 38 weeks of gestation by cesarean section at a tertiary care hospital, was healthy at birth. There was no history of varicella, German measles, or any viral infection during pregnancy, especially the TORCH complex (Toxoplasma, Zika, syphilis, rubella, Cytomegalovirus, herpes). The mother received two doses of tetanus at 16 and 20 weeks of gestation, one dose of Boostrix (diphtheria, tetanus, and acellular pertussis) at 28 weeks, and one dose of influenza vaccine (Influvac) at 28 weeks. The birth weight of the baby was 3450 grams, the length was 52 cm, and the orbitofrontal circumference (OFC) was 36 cm. The Apgar score is a score given to newborns based on the baby's heart rate, muscle tone, skin color, respirations, and reflex irritability with a range of 0 to 10. A score of 7-10 is reassuring, 4-6 is moderately abnormal, and 0-3 requires immediate attention. The baby's Apgar score was 9, 10, and 10 at one, five, and 10 minutes, respectively. The baby was apparently alright; however, the parents noticed engorged blood vessels and distinctive patterns of discoloration and dilation of blood vessels on the left leg (Figure 1).

Most of the tests conducted on heel prick blood were within normal limits, including complete blood count (CBC), protein C and S, and thyroid profile. Serum calcium was slightly less than normal and liver function tests (LFT) indicated elevated aspartate aminotransferase (AST), lactate dehydrogenase (LDH), and international normalized ratio (INR) (Table 1).

**Table 1:** Lab investigation reports on the heel prick blood sample. CBC: complete blood count; WBC: white blood cells; INR: international normalized ratio; aPTT: activated partial thromboplastin time; A:G ratio: albumin-to-globulin ratio; SGOT/AST: serum glutamic-oxaloacetic transaminase/aspartate aminotransferase; SGPT/ALT: serum glutamic-pyruvic transaminase/alanine aminotransferase; GGT: gamma-glutamyl transferase; LDH: lactate dehydrogenase; TSH: thyroid-stimulating hormone; T3: triiodothyronine; T4: thyroxine.

Sr.	CBC	Result	Biological reference interval
1	Hemoglobin	16.3 gm/dL	Birth: 14-22 gm/dL
2	Total WBC count (neutrophils, lymphocytes, monocytes, eosinophils, and basophils)	15600/cu.mm (67%, 26%, 5%, 2%, and 0%)	Birth: 1000-26,000/cu.mm
3	Platelet count	305000/cu.mm	At birth: 1.0-4.5 lakhs/cu.mm
4	Prothrombin time/prothrombin ratio	18.96 sec/1.46	11-15 sec
5	INR	1.52	1-1.5
6	aPTT	29.8 sec	30-40
7	ABO group (tube & slide agglutination)	O positive	-
8	Liver function test (bilirubin total = direct + indirect)	4.99 mg/dL (0.00+4.99)	Term neonate: <10 mg/dL (direct: 0.0-0.6 mg/dL, indirect: 0.6-10.5 mg/dL)
9	Total protein	7.15 gm/dL	Children: 6.6-8.0 gm/dL
10	Albumin	4.11 gm/dL	Children: 3.8-5.4 gm/dL
11	Globulin	3.04 gm/dL	-
12	A:G ratio	1.3	-
13	SGOT (AST)	216 U/L	Male: 17-59 U/L
14	SGPT (ALT)	13 U/L	Male: 21-72 U/L
15	Alkaline phosphatase	522 U/L	Neonate: 73-391 U/L, infant: 59-425 U/L
16	Serum GGT	143 U/L	Cord blood: 37-193, 0-1 month: 13-147
17	Serum LDH	733 U/L	Newborn: 160-450 U/L, infant: 100-250 U/L
18	Serum calcium (Endpoint Arsenazo III)	8 mg/dL	Newborn: 9-10.6 mg/dL, children: 8.8-10.8 mg/dL
19	Thyroid profile: TSH	9.27 µIU/L	Term infant TSH: 1.0-38.9 µIU/L
	T3	1.21 ng/ml	Cord blood T3: 0.3-0.7 ng/ml, newborn T3: 0.75-2.6 ng/ml
	T4	11.62 µg/dL	1-3 days T4: 8.2-19.9 µg/dL, 1 week T4: 6.0-15.9 µg/dL
20	Protein C functional activity	32.3	28-54
21	Protein S functional activity	39%	Day 1: 28-47%, day 3: 33-67%, up to 1 year: 29-162

A Doppler of the femoral artery and vein showed normal triphasic flow and waveforms without any evidence of significant luminal stenosis. There was also a lower limb length discrepancy of 1.5 cm. Family history revealed that the couple had another daughter aged three years with a small hemangioma on her left cheek. Genetic testing using fluorometric enzyme immunoassay screening revealed a negative screening report (Table 2).

**Table 2:** Genetic screening report using fluorometric enzyme immunoassay. This table represents the values corresponding to the results' values. TSH values are represented in the serum unit (conversion factor: 1 µU/mL blood = 2.22 µU/mL serum). NMT: not more than; NLT: not less than; 17-OHP: 17α-OH-progesterone; G6PD: glucose-6-phosphate dehydrogenase; IRT: human immunoreactive trypsinogen; BTD: profound biotinidase; T GAL: total galactose (galactose and galactose-1-phosphate); TSH: thyroid-stimulating hormone; PHE: phenylalanine.

Sr. No.	Disorder	Parameter	Result	Reference	Remarks
1	Congenital adrenal hyperplasia	17-OHP	8.1	NMT 65 nmol/L	Normal
2	Glucose 6 phosphate dehydrogenase deficiency	G6PD	43.4	NLT 3 U/dL	Normal
3	Cystic fibrosis	IRT	25.8	NMT 90 ng/mL	Normal
4	Profound biotinidase deficiency	BTD	175.3	NLT 15 U/dL	Normal
5	Classical galactosemia	T GAL	13.8	NMT 25 mg/dL	Normal
6	Congenital hypothyroidism	TSH	2.219	NMT 25 µIU/mL	Normal
7	Phenylketonuria	PHE	82.71	NMT 169 µM/L	Normal

Otologic screening using distortion product otoacoustic emissions (DPOAEs) revealed normal functioning of outer hair cells in both ears, and the doctors advised aural hygiene. DPOAE is a tool used for hearing assessment and scientific study. They are produced by the nonlinear interaction of two tones within the cochlea, which is due to the normal function of outer hair cells (OHCs). DPOAEs can be used to detect hearing loss with accuracy, and are one of the most common otoacoustic emissions recorded in clinical practice.

The case was diagnosed as a case of CMTC, after ruling out all genetic causes of such lesions, like congenital adrenal hyperplasia, glucose 6 phosphatase dehydrogenase (G6PD) deficiency, cystic fibrosis, profound biotinidase deficiency, classical galactosemia, congenital hypothyroidism, phenylketonuria, etc. It was not associated with any other significant health problems. In this case, no specific treatment was required and the condition improved with time over months of follow-up. The parents were counseled that there is no cure for CMTC, and treatment primarily focuses on managing symptoms and potential complications, which involves close monitoring, addressing any related health issues, and providing supportive care.

## Discussion

A few hundred cases of CMTC have been reported in the literature from around the world, with only a few from the Indian sub-continent [2,3]. The current case is the first male baby with CMTC in eastern India. Two cases have been reported from east India (West Bengal); one in an 11-year-old girl and another in a full-term newborn female baby. The former had bilateral involvement and truncal sparing along with hypoplasia of toes without limb-length discrepancy, with angiokeratoma at 10 years of age [4]. However, there was a history of consanguinity. The latter case involved the whole body with ulceration over the extensor aspects of both the knee joints and the right elbow joint [5]. A case from northern India (Himachal Pradesh) was reported with involvement of the hand, arms, legs, and a portion of the chest, but with no other congenital anomaly [6]. Another case was reported from south India in an 80-day-old female child who presented with lesions involving the left upper and lower limbs with underlying atrophic changes, seen over the left extremities. A case was reported from western India (Maharashtra) in a 20-year-old female patient with lesions since childhood along with ulcerations on both breasts [7]. A second case from the same region was reported in a 24-year-old male who presented with congenital multiple reddish and bluish mesh-like skin lesions all over the body, with left lower leg phlebectasia [8]. A third case was reported from central India (Sevagram) with reticular erythematous patches over the right thigh and anterolateral aspect of the right side of the chest at the time of birth. Many cases deficient in proteins C and S have been described and have an associated thrombotic tendency [9], but no such deficiency was present in the current case. However, there were slight elevations in LFTs, especially AST and LDH, indicating liver damage. Previous studies have also shown liver abnormalities in nearly 10% of the babies with CMTC [10]. A case of a 41-year-old female from the western coast (Gujarat) was reported along with hypothyroidism and ophthalmic lesions showing bilateral peripheral retinal vascular abnormalities, peripheral retinal nonperfusion on fluorescein angiography, and bilateral optic disk drusen [11]. A second case from the same region in a two-month-old boy was reported with widespread lateralized blue macules (nevus cesius), an extensive nevus flammeus, and large patches of CMTC. Moreover, it was also associated with macrocephaly and other defects [12]. The current case from eastern India did not have any history of consanguinity or any other congenital defects. Counseling of the parents is important to help them understand the difference in treatment modalities based on clinical features, i.e., CMTC with no congenital defects versus CMTC with other congenital defects.

## Conclusions

The current case is the first male baby with CMTC in eastern India, without any history of consanguinity or thrombotic tendency with slight limb length discrepancy and liver function abnormalities. The otological screen and genetic testing were also normal. The case had a good prognosis and the lesions improved over six months of follow-up. This case has implications for clinical practice as it was non-progressive and resolved in six months without any intervention. Hence, not all congenital malformations warrant further management and simple counseling and assurance can allay the fear and anxiety of parents.
